# Improving Electrochemical Performance of Ultrahigh-Loading Cathodes via the Addition of Multi-Walled Carbon Nanotubes

**DOI:** 10.3390/nano15030156

**Published:** 2025-01-21

**Authors:** Chan Ju Choi, Tae Heon Kim, Hyun Woo Kim, Do Man Jeon, Jinhyup Han

**Affiliations:** 1 Department of Chemical Engineering, Keimyung University, Daegu 42601, Republic of Korea; 2 Research Center for Materials Analysis, Korea Basic Science Institute (KBSI), Daejeon 34133, Republic of Korea; 3 Gumi Electronics & Information Technology Research Institute (GERI), Gumi 39171, Republic of Korea

**Keywords:** high-nickel cathode, multi-walled carbon nanotubes, ultrahigh-loading cathodes

## Abstract

Achieving high energy densities in lithium-ion batteries requires advancements in electrode materials and design. This study investigated the incorporation of multi-walled carbon nanotubes (MWCNTs) with high commercial viability as conductive additives into two types of high-nickel cathode materials, LiNi_0.8_Co_0.1_Mn_0.1_O_2_ and LiNi_0.92_Co_0.07_Mn_0.01_O_2_. To ensure a uniform distribution within the electrodes, MWCNTs were uniformly dispersed in the solvent using ultrasonication, the most effective and straightforward dispersion method. This enhancement improved both electronic and ionic conductivity, facilitating the formation of an efficient electron transfer network. Unlike the cells using only carbon black, the electrodes with MWCNTs exhibited lower internal resistances, facilitating higher lithium-ion diffusion. The cells with MWCNTs exhibited a capacity retention of 89.5% over their cycle life, and the cells with 2 wt% MWCNTs exhibited a superior rate capability at a high current density of 1 C. This study highlights that incorporating well-dispersed MWCNTs effectively enhances the electrochemical performance of ultrahigh-loading cathodes in lithium-ion batteries (LIBs), providing valuable insights into electrode design.

## 1. Introduction

With the rapid growth of lithium-ion batteries (LIBs) applications in electric vehicles (EVs) and energy storage systems (ESS), cathode materials with high energy densities and long cycle lives are required [1,2]. Among the potential candidates, LiNiO_2_ (LNO), a cathode material with 100% Ni content, has gained widespread attention from researchers owing to its suitability for high-energy-density applications [3,4]. Concurrently, the development of LiNi_x_Co_y_Mn_z_O_2_ (NCM) cathode compositions has evolved from early formulations, such as LiNi_0.33_Co_0.33_Mn_0.33_O_2_ (NCM111) to mid-Ni variants, such as LiNi_0.5_Co_0.2_Mn_0.3_O_2_ (NCM523) and LiNi_0.6_Co_0.2_Mn_0.2_O_2_ (NCM622), advancing to high-Ni compositions, such as LiNi_0.8_Co_0.1_Mn_0.1_O_2_ (NCM811) [5,6]. An increase in the Ni content in high-Ni cathodes presents challenges in reducing Co and Mn, particularly influencing the thermal and structural stabilities [7]. These factors contribute to the decreased cycle retention owing to volume expansion of the electrode and the formation of spinel-like structures [8]. In addition, higher Ni levels lower the thermal stability, leading to increased oxygen release and accelerated phase transitions that weaken the structural integrity of materials [7]. To address these issues, there has been significant interest in balancing the maximization of the Ni content and ensuring stability. Given these considerations, materials such as LiNi_0.8_Co_0.1_Mn_0.1_O_2_ (NCM811) and LiNi_0.92_Co_0.07_Mn_0.01_O_2_ (Ni92%), which combine increased Ni levels with structural integrity, are emerging as crucial solutions for enhancing performance and stability.

High-Ni cathodes are essential to achieve high energy density. Advancements in electrode engineering are essential to unlock their full potential. Ultrahigh-loading electrodes, characterized by an increased active material mass per unit area, play a critical role in maximizing energy density. By efficiently utilizing the limited device space, these electrodes contribute to substantial improvements in overall energy storage [9]. Although ultrahigh-loading electrodes are advantageous, they present inherent challenges that require effective solutions to achieve their full performance potential.

The intrinsic issues and challenges associated with ultrahigh-loading electrodes are as follows:

1. Volumetric variations;
2. Limited electrolyte diffusion and electron transfer;
3. Increased resistance.

One of the critical challenges is the severe volumetric variations experienced by the active materials during the metal ion insertion and extraction processes [10]. These dynamic microstructural changes generate internal micro strains that can undermine the stability of the solid electrolyte interphase (SEI). Unstable SEI formation adversely affects the cycling performance of high-load electrodes, leading to reduced long-term reliability [11,12]. Additionally, thicker electrode layers in high-loading electrodes may lead to limited electrolyte diffusion efficiency and restricted electron transfer depth [9]. These limitations increase the total resistance of the electrode, reduce the electronic conductivity, and hinder efficient electrochemical reactions, posing significant challenges for maintaining high performance over extended cycles [13]. The integration of multi-walled carbon nanotubes (MWCNTs) into the electrode structure can mitigate these issues by enhancing electronic conductivity and providing additional structural benefits. 

Table 1 summarizes the previous studies on the use of not only multi-walled carbon nanotubes (MWCNT) but also various carbon conductive materials at a high-loading level. Since the early 2000s, various carbon materials such as carbon black (CB), single-walled carbon nanotubes (SWCNTs), double-walled carbon nanotubes (DWCNTs), MWCNTs, graphene, graphite, and carbon fibers have been investigated as conductive additives in electrodes. By 2005, graphite was mixed with carbon black as a conductive material for cathode [14]. In particular, CB has been widely utilized due to its low cost. However, the spherical particle shape of CB requires a large quantity when used in electrodes, which decreases the energy density of lithium-ion batteries. Carbon nanofibers, with their rod-like structure, form robust conductive pathways, enabling electron distribution to the active material within cathodes. However, their elongated structure reduces the contact area, limiting their standalone use [15,16].

**Figure 1:**
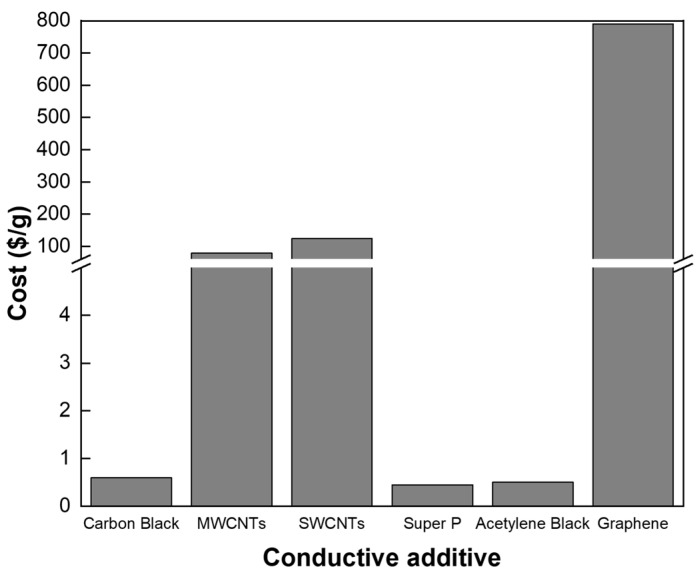
Comparison of various types of conductive additives for electrode fabrication and their costs per gram.

In the case of MWCNTs, they offer several advantages, including high theoretical electrical conductivity and an extremely high aspect ratio [22]. These characteristics enable long-range electron transport and enhance conductivity effectively with minimal additive content, helping to overcome the conductivity limitations of thick electrodes [23]. As shown in Figure 1, CNTs-based materials (Sigma Aldrich, St. Louis, MO, USA) are approximately 130–200 times more expensive than carbon black (Beyond Battery, Singapore), but they are less expensive than SWCNTs and graphene. While MWCNTs are relatively high priced compared to other carbon materials, they offer high competitiveness when used in minimal amounts. Considering the balance of cost, performance, and scalability, MWCNTs are highly promising for commercialization while effectively increasing energy density.

Although MWCNTs offer considerable advantages, they encounter key challenges that limit their practical applications. Achieving a homogeneous distribution of MWCNTs presents another challenge. This is because the high aspect ratio of MWCNTs leads to entanglement and bundling, making their dispersion particularly challenging and presenting significant hurdles for their effective utilization [24,25].

To prevent agglomeration, which is triggered by the strong van der Waals interactions between MWCNTs, ultrasonication is used as a dispersion method [26]. Ultrasonication technology has the advantage of simplifying process complexity. Moreover, it does not generate waste water or cause significant environmental pollution, making it highly suitable for scalability in commercial applications. Previous studies in areas other than battery technology, such as nanofluids and graphene quantum dots, have successfully demonstrated the effectiveness of ultrasonication in enhancing colloidal stability and facilitating the synthesis of homogeneously dispersed nanomaterials [27,28]. Ultrasonication has proven to be effective in breaking up particle clusters and achieving uniform dispersion at the nanoscale. This approach has also been used in batteries, particularly with NCM622 and NCM811 cathode materials, demonstrating that the optimized dispersion of MWCNTs via ultrasonication enhances the electron transfer network in the electrode, leading to enhanced performance at high current densities [20,29]. The study primarily focused on the NCM811 cathode with a loading level of approximately 7 mg/cm^2^. To further increase the energy density, it is essential to increase the Ni content and the loading level of cathodes.

In this study, we investigated the use of high-Ni cathodes (NCM811, Ni92%) with an ultrahigh loading level (>11.2 mg/cm^2^) to improve cycle performance. By incorporating minimal amounts of MWCNTs (1 and 2 wt%) into carbon black, we aimed to enhance the energy density, cycling performance, and conductivity of the electrode under ultrahigh-loading conditions. Ultrasonication was applied to ensure the effective dispersion and uniform distribution of the MWCNTs in the electrode. The physical and chemical properties of the electrodes were analyzed using scanning electron microscopy (SEM) and X-ray diffraction (XRD). The electrochemical performance was evaluated using galvanostatic charge/discharge tests, electrochemical impedance spectroscopy (EIS), and rate capability evaluations.

## 2. Experimental

### 2.1. Characterization of Materials

Commercial LiNi_0.8_Co_0.1_Mn_0.1_O_2_ (NCM811) and LiNi_0.92_Co_0.07_Mn_0.01_O_2_ (Ni92%) cathodes were used as active materials. SEM (JSM-IT500, JEOL, Tokyo Japan at 10 kV) was used to observe the morphologies and particle sizes of active materials. Cross-sectional images of the internal structures of the fabricated electrodes were obtained using a focused ion beam (Crossbeam 550, ZEISS, Oberkochen, Germany, at 5 kV).

The XRD patterns of the cathode materials were obtained on a Multi-Purpose X-ray diffractometer (Malvern Panalytical, Almelo, The Netherlands) using a Cu-Kα X-ray source (λ = 1.5406 Å) in the 2θ range of 10–80°.

### 2.2. Preparation of Electrodes

Commercially available MWCNTs (3–15 nm; purity > 97%) were immersed in *N*-methyl-2-pyrrolidone (NMP, Sigma Aldrich, St. Louis, MO, USA) and used as the organic solvent to prepare a dispersion containing 5 wt% MWCNTs in the solid content. The MWCNTs in the solvent were dispersed by ultrasonication (VC-505, SONICS & Materials, Newtown, CT, USA at 10 kHz) for 2 h. Carbon black (C45, Imerys Graphite & Carbon, Bodio, Switzerland) was used as a conductive additive. The binder used in the cathode preparation was polyvinylidene difluoride (Solvay, Brussels, Belgium) in the form of 8 wt% solution in NMP. 

The compositions of the prepared electrodes are summarized in Table 2. The active materials, conductive additives, and binders were mixed in a centrifuge mixer (ARE-310, THINCKY MIXER, Tokyo, Japan). For ultrahigh loading, the slurry of cathode materials was cast onto an Al foil current collector with a consistent thickness of 300 µm using a doctor blade. After that, it was dried in an oven at 80 °C for 1 h, then in a vacuum oven at 90 °C for 24 h. The mass loading values were measured on average to be ~16 (0 wt%), 14 (1 wt%), and 11 mg/cm^2^ (2 wt%).

### 2.3. Cell Assembly and Electrochemical Measurements

The cathodes were assembled in an Ar-filled glovebox in a CR-2032 coin-type cell, with Li metal as the anode. Commercial 1 M LiPF_6_ in EC:EMC (3:7 weight ratio) electrolyte (ENCHEM Co., Ltd., Jecheon, Republic of Korea) and a separator were used to conduct electrochemical performance tests in a 30 °C chamber for charge/discharge cycling (CT-4008Tn, NEWARE, Shenzhen, China).

The prepared coin cells were analyzed using a galvanostatic rate capability test in the voltage range of 2.8–4.3 V (V vs. Li^+^/Li^0^). The charge and discharge current densities for rate capability varied from 0.1 to 1 C (1 C = 200 mA g^−1^) at each rate for three cycles. EIS was performed in the frequency range of 0.1 MHz–100 mHz with a sinus amplitude of 5 mV using an electrochemical workstation (SP-200, Biologic, Seyssinet-Pariset, France).

## 3. Results and Discussion

### 3.1. Characterization of Cathode Materials

**Figure 2:**
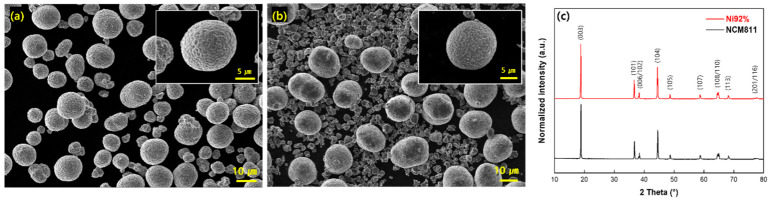
SEM image of (**a**) commercial LiNi_0.8_Co_0.1_Mn_0.1_O_2_ (NCM811) cathode and (**b**) commercial Ni92% cathode. (**c**) XRD patterns of high-Ni cathodes (NCM811, Ni92%).

Figure 2 shows the SEM images of commercial NCM811 and Ni92% cathodes at different magnifications. All the powders had a spherical morphology, and the secondary particles were densely composed of primary particles. Both high-Ni cathode materials exhibited a bimodal particle size distribution of the secondary particles. Large NCM811 particles measured approximately 14 μm, while Ni92% measured approximately 16 μm. The size of the smaller secondary particles of NCM811 ranged from 5 to 9 μm, while that of Ni92% ranged from 2 to 3 μm. The tap densities of cathodes were measured to be 1.88 and 2.51 g/cm^3^ for NCM811 and Ni92%, respectively. The reason for the difference in tap densities of NCM811 and Ni92% is the greater distribution of relatively small particles in the bimodal state, as observed in the SEM images (Figure 2a,b).

The XRD patterns of the NCM811 and Ni92% cathodes are shown in Figure 2c. The NCM811 and Ni92% cathodes exhibited a well-defined layered hexagonal α-NaFeO_2_ structure, belonging to the *R*$\bar{3}$m space group. The evident split peaks at (006)/(102) and (108)/(110) indicate a well-defined layered structure in both cathodes. No impurities or secondary phases were observed.

### 3.2. Characterization of Electrode Incorporated with MWCNTs

**Figure 3:**
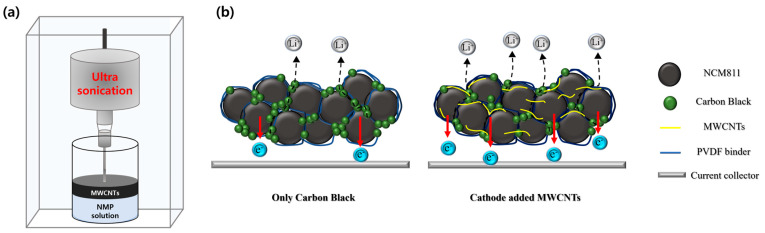
(**a**) Schematic of ultrasonication for the preparation of MWCNT dispersion solution. (**b**) Schematic comparison of internal Li^+^ and electron transport behavior of electrodes with only carbon black and inserted MWCNTs.

To enhance the electrochemical performance of ultrahigh-loading cathodes, it is crucial to address the issue of low conductivity in electrodes, particularly the thick electrodes. High-loading cathodes face several challenges, such as volumetric variations, limited electrolyte diffusion and electron transfer, and increased resistance. Incorporating MWCNTs into the electrode composition enhanced the electrical conductivity, leading to reduced internal resistance. However, the primary factor hindering the utilization of MWCNTs is their dispersion [30]. Owing to the strong van der Waals forces, MWCNTs tend to aggregate readily, forming bundles that obstruct their uniform dispersion in slurries containing active materials and binders [26]. To overcome these issues, an ultrasonic treatment was performed using MWCNTs as a conductive additive. A normal disperser and ultrasonication were used as dispersion methods; however, the higher power of ultrasonication resulted in a more homogenous dispersion. Images of the dispersed solutions obtained using each technique are shown in Figure S1 in the Supplementary Materials. In addition, ultrasonication treatment is a relatively simple technique for preparation of MWCNTs dispersion and is scalable, making it highly suitable for commercial applications. Therefore, only ultrasonication was used for electrode preparation to consider scalability and commercial feasibility in future applications. Figure 3a shows the scheme for the preparation of MWCNTs in NMP using an ultrasonic processor. NMP was selected as the organic solvent because of its Lewis basicity and highly polar molecular structure, which facilitate the dispersion of MWCNTs [31]. These properties facilitate the NMP molecules to form strong bonds with the CNTs, promoting effective dispersion and preventing agglomeration. A solid probe was immersed in the MWCNT-containing solution, and physical treatment was performed using ultrasonication. The ultrasonication process dispersed the agglomerated carbon particles, which were then incorporated into the electrode slurry.

Two types of high-Ni cathode materials, NCM811 and Ni92%, were used, and carbon black and well-dispersed MWCNTs were used as the conductive materials. Figure 3b shows a schematic of the ion and electronic conduction on the electrode surface. Figure 3b highlights the differences in the internal Li-ion and electron transport behaviors of the two electrodes. The increased electrode thickness in ultrahigh-loading cathodes extends the Li-ion pathways, reducing the electronic conductivity. MWCNTs, well known for their excellent electrical conductivity (10^6^–10^7^ S cm^−1^), exhibit higher conductivity than carbon black (0.1–10^2^ S cm^−1^) and form effective connections between the active materials and carbon black [32,33]. The MWCNTs were surrounded by secondary particles and carbon black nanoparticles. The addition of highly conductive MWCNTs led to the formation of a 3D electrical network, facilitating efficient ion and electron transport, with Li ions moving rapidly and in greater quantities [34]. 

**Figure 4:**
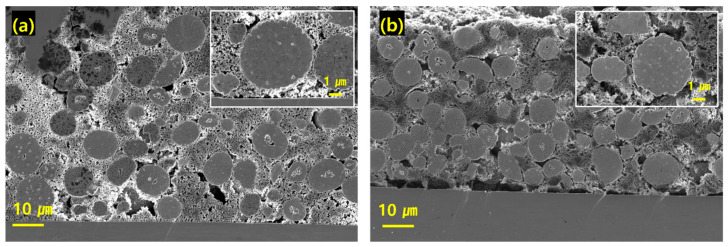
(**a**) SEM image of internal structure of pristine NCM811 electrode; inset: spherical active material particles surrounded by carbon black. (**b**) SEM image of internal structure of NCM811 electrode incorporated with 2 wt% MWCNTs; inset: spherical active material particles in conductive additive containing MWCNTs.

Ion milling of electrodes was conducted to examine the internal structure of the high-load cathode. Figure 4a shows the cross-sectional images of the NCM811 electrode, where the thickness of the electrode using only carbon black is measured to be approximately 68 µm. Figure 4b shows the electrode with 2 wt% MWCNTs, with a thickness of approximately 60 µm, with carbon evenly distributed around the active material under ultrahigh loading. The uniform distribution of the conducting additive around the active material plays a crucial role in enhancing electrical conductivity and mechanical integrity. This finding helps to maintain the electrochemical stability of the electrode surface during cycling. The surface morphologies of the electrodes after cycling are shown in Figure S2 in the Supplementary Materials. The cracks in the electrode containing MWCNTs were smaller than those in the pristine electrode. In addition, Figures S3 and S4 show energy-dispersive X-ray spectroscopy mapping of the NCM811 and Ni92% electrodes, indicating that the active material and carbon components are well integrated across various regions of the cathode. Elemental mapping images of the distributed transition metals revealed the microstructure and chemical composition of the pristine and 2 wt% MWCNT-containing electrodes before cycling. The elemental distributions of Ni, Co, Mn, and C of the conductive additive were uniform in both electrode types using the two cathode materials. Additionally, SEM images were taken to examine the distribution of MWCNTs within the electrodes. As seen in Figure S5, more wires were found in the electrodes containing 2 wt% MWCNTs, both with NCM811 and Ni92% cathode materials. These wires mixed with the active materials and carbon blacks confirmed that good dispersion was achieved.

### 3.3. Electrochemical Properties of Li Half-Cells Depending on Varying MWCNTs Content

**Figure 5:**
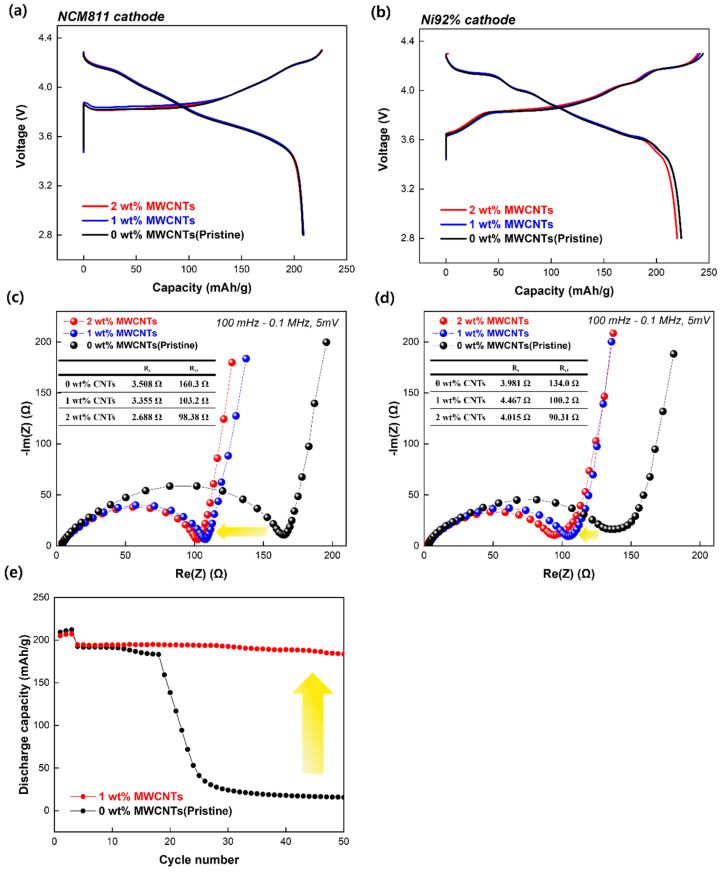
(**a**) Initial voltage profile at 0.1 C between 2.8 and 4.3 V vs. Li using NCM811 and (**b**) Ni92% cathode. EIS profiles of the Li half-cell with 0, 1, and 2 wt% MWCNTs using the (**c**) NCM811 and (**d**) Ni92% cathode. (**e**) Cycling performances of electrodes prepared with ratio of 88:2:10 (active material:conductive additive:binder material) in Li half-cell at 0.33 C (first three cycles at 0.1 C) between 2.8 and 4.3 V vs. Li.

The electrochemical performances were compared based on the voltage profiles of cells cycled in a Li half-cell at a current density of 0.1 C in the voltage range of 2.8–4.3 V vs. Li^+^/Li^0^. Figure 5a shows the initial voltage profiles of the Li half-cells using the pristine electrode and those incorporating 1 and 2 wt% MWCNTs in the NCM811 cathode. The discharge capacities of the cells were 208.7, 209.0, and 208.3 mAh g^−1^ for the 0, 1, and 2 wt% MWCNTs, respectively. No significant difference in the initial discharge capacity was observed with respect to MWCNT content. The NCM811 cathode exhibited IR drop during the initial charge process, which appeared to be consistent across all samples, suggesting that it was a temporary resistance caused by the increased thickness of the ultrahigh-loading electrode. Figure 5b shows the discharge capacities of the Ni92% cathode, which were 223.7, 223.5, and 219.7 mAh g^−1^ for the electrodes containing 0, 1, and 2 wt% MWCNTs, respectively. The initial capacities indicated that the MWCNTs had minimal influence on the initial low current rate. In addition, the Ni92% cells exhibited higher discharge capacities than the NCM811 cells owing to their higher Ni contents. 

To further investigate the influence of the MWCNTs on the half-cells, EIS was conducted to evaluate the impedance of the electrode. EIS spectra represent the Nyquist plots generated utilizing a frequency range of 0.1 MHz–100 mHz before cycling. The EIS spectra showed a semicircle in the high-frequency range and a straight line in the low-frequency range. Figure 5c shows the internal resistance of the electrode using the cathode material NCM811 with varying MWCNT content. The resistances of the electrolyte solutions (R_s_) are nearly the same. Charge transfer resistances (R_ct_) before cycling were determined to be 160.3, 103.2, and 98.38 Ω for the electrodes prepared with 0, 1, and 2 wt% MWCNTs, respectively. As shown in Figure 5d, the cells with the Ni92% cathode do not exhibit any difference in R_s_. However, the R_ct_ values for the 0, 1, and 2 wt% MWCNTs were 134.0, 100.2, and 90.31 Ω. For both cathode materials, the pristine cell exhibited significantly higher resistance to charge transfer. The cells containing 2 wt% MWCNTs exhibited the lowest R_ct_, indicating enhanced electrical conductivity. The yellow arrow indicates that the addition of MWCNTs reduced the resistances within the electrode. The improvement in charge transfer could be attributed to the conductive network of the MWCNTs, which facilitated more efficient ion and electron transport between the active material particles and electrolytes, leading to superior cycle retention of the high-loading cathode. The addition of MWCNTs confirmed that both cathode materials resulted in a decrease in total cell resistance.

Figure 5e shows the cycling performance of the pristine and 1 wt% MWCNT electrodes fabricated using 88 wt% active material and 2 wt% conductive additives. The cycle performances were tested at 0.1 C for the 3 cycles and 0.33 C for the next 47 cycles, between 2.8 and 4.3 V. The 50th discharge capacity of the electrode with MWCNTs was 183.7 mAh g^−1^. In contrast, the pristine electrode exhibited a substantial decrease in capacity before reaching 20 cycles, with a 50th-cycle capacity of only 15.7 mAh g^−1^, demonstrating poor cycling performance. The capacity retentions of the electrodes were 89.5% (1 wt% MWCNTs) and 7.5% (pristine electrode) for up to 50 cycles. Capacity retention is affected by the presence or absence of MWCNTs, as indicated by the yellow arrow. The conductive network was insufficiently formed for the electrode containing only carbon black, resulting in sudden capacity loss. In contrast, the incorporation of MWCNTs connects the active materials, forming an efficient and hybrid conductive network that helps mitigate capacity degradation. This result indicated that the addition of MWCNTs to the electrode resulted in enhanced capacity retention of the discharge capacity and more reversible behavior compared to cells without MWCNTs.

**Figure 6:**
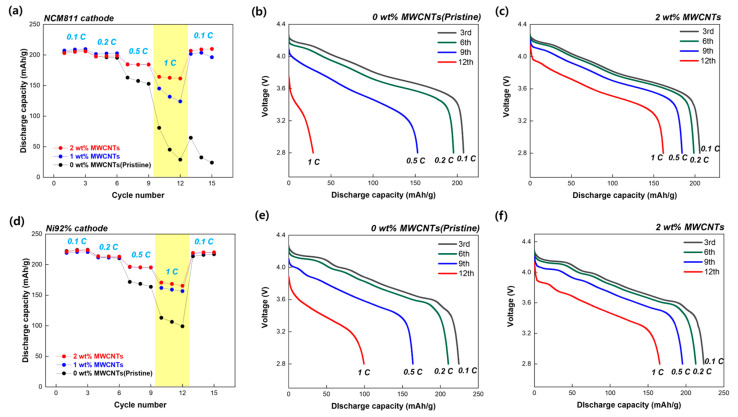
(**a**) Rate capability of the Li half-cell with NCM811 from 0.1 to 1 C between 2.8 and 4.3 V. (**b**) Discharge capacity curves of pristine electrode at various current rates and (**c**) discharge capacity curves of NCM811 containing 2 wt% MWCNTs. (**d**) Rate capability of Li half-cell with Ni92% from 0.1 to 1 C between 2.8 and 4.3 V. (**e**) Discharge capacity curves of pristine electrode at various current rates and (**f**) discharge capacity curves of Ni92% containing 2 wt% MWCNTs.

In addition to the cycling stability and internal resistance, the rate capability of the electrodes was analyzed to evaluate their electrochemical performance under various rate conditions. Owing to recent issues related to the fast charging of EVs, the C-rate performance of cathodes has become a crucial factor in the commercialization of batteries. Figure 6a shows the rate capabilities of the NCM811 cathodes with various MWCNT contents. Compared with the cells incorporated with MWCNTs, the pristine electrode exhibited a substantial decrease in capacity at high current rates and low recovery. Particularly at a C-rate of 1 C for NCM811, the specific capacities for 0, 1, and 2 wt% MWCNTs were 28.9, 124.1, and 161.6 mAh g^−1^, respectively. As shown in Figure 6b,c, the discharge capacity at 1 C compared to 0.1 C is only 14% for 0 wt% MWCNTs, while it reaches 78.5% for 2 wt% MWCNTs. MWCNTs are expected to form a 3D network that maintains electrical contact with the active material during a high charge/discharge rate and exhibits recoverability when transitioning to a low current rate. Figure 6d shows the rate capability of the Ni92% cathodes as a function of the MWCNTs content. Owing to the high active material ratio, the pristine electrode experienced capacity degradation at 1 C, whereas the cells with 1 and 2 wt% MWCNTs maintained a higher capacity than the pristine cell as indicated in yellow area. Figure 6e,f show that the capacity retention at 1 C in the cell using Ni92% is 44.2% for 0 wt% MWCNTs and 74% for 2 wt% MWCNTs. This finding contributed to improved kinetics, leading to a higher capacity, particularly at higher current rates. Consequently, an effective 3D conductive network helped to achieve superior rate capability, with 2 wt% MWCNT-containing NCM811 and Ni92% contributing to excellent electrochemical performance. In summary, the addition of MWCNTs under ultrahigh-loading conditions facilitated a long life and good capacity retention at a high current rate.

## 4. Conclusions

In this study, ultrahigh-loading cathodes were prepared using NCM811 and Ni92% to enhance energy density and electrochemical performance of LIBs. Considering cost and process complexity, scalable MWCNTs were selected as the conductive carbon material. The incorporation of MWCNTs as conductive additives, dispersed uniformly through ultrasonication, established an effective conductive network in the electrodes, leading to improved cycle life and rate capability of the Li half-cell. The electrochemical properties of the NCM811 and Ni92% cathodes were investigated by varying the MWCNT content (0–2 wt%). In the cell with 88 wt% active material of NCM811 and 2 wt% conductive additive, the capacity retention of the cell with MWCNTs (89.5%) was over 10 times higher than that of the pristine cell (7.5%). At a current rate of 1 C, the 2 wt% MWCNT cells demonstrated a significantly higher capacity than the pristine cells, highlighting the superior electrochemical performance of the MWCNTs under high-loading conditions. The effects of MWCNTs were particularly pronounced at high current rates, which was attributed to the lower resistances in the cells containing MWCNTs, as measured using EIS.

This study demonstrated that the addition of minimal amounts of MWCNTs to ultrahigh-loading electrodes ensured long-range electron transport, contributing to enhanced capacity retention. These findings will contribute to the future design of ultrahigh-loading cathodes for LIBs, providing valuable insights into the development of high energy density from a process engineering perspective.

## Figures and Tables

**Table 1:** Summary of previous studies.

#	Year	Cathode	Mass Loading Level (mg/cm^2^)	Conductive Additive	C-Rate	Ref
1	2005	LiFePO_4_	3.0~4.9	CB/graphite	0.5 C	[14]
2	2008	LiCoO_2_	-	CB/carbon fibers/MWCNTs	2 C	[15]
3	2016	NCM622	10	CB/carbon fibers	1 C	[16]
					0.1–10 C	
4	2017	NCM111	2.27	CB/CNTs/graphene nanosheets	0.2–3 C	[17]
5	2019	NCM523	6.5	SWCNTs	1 C	[18]
					0.2–10 C	
6	2023	LiFePO_4_	~8.4	SW/DW/MW CNTs	1 C	[19]
					0.1–10 C	
7	2023	NCM811	~7	CB/CNTs	1 C	[20]
8	2024	NCM622	10.5~11.5	CB/CNTs	1 C	[21]
					0.1–10 C	
9	2025	NCM811, Ni92%	11~16	CB/MWCNTs	0.1–0.33 C	This work
					0.1–1 C	

**Table 2:** Composition of all electrodes in this study.

Sample Name	Types of Cathode	Active Materials (wt%)	Conductive Additive (wt%)		Binder Materials (wt%)
			Carbon Black	MWCNTs	
0 wt% MWCNTs	NCM811	88	8	**0**	4
1 wt% MWCNTs		88	7	**1**	4
2 wt% MWCNTs		88	6	**2**	4
0 wt% MWCNTs	Ni92%	88	8	**0**	4
1 wt% MWCNTs		88	7	**1**	4
2 wt% MWCNTs		88	6	**2**	4

## Data Availability

The data presented in this study are available on request from the corresponding author.

## Supplementary Materials

The following supporting information can be downloaded at: https://www.mdpi.com/article/10.3390/nano15030156/s1, Figure S1: Comparison of MWCNTs dispersion using normal sonication and ultrasonication. MWCNTs are homogenously distributed through ultrasonication; Figure S2: Surface images of electrodes after conducting C-rate capability test, performing recovery from high current rate of 1 C to lower rate of 0.1 C for (a) pristine cathode and (b) MWCNT-incorporated cathode; Figure S3: Energy dispersive spectroscopy mapping images of NCM811 cathode metals in the internal structure of the (a) electrode containing only carbon black and (b) electrode incorporating 2 wt% MWCNTs; Figure S4: Energy dispersive spectroscopy mapping images of Ni92% cathode metals in the internal structure of the (a) electrode containing only carbon black and (b) electrode incorporating 2 wt% MWCNTs; Figure S5: Images of inside the electrode with NCM811 (a) pristine electrode and (b) electrode with 2 wt% MWCNTs. Images of inside the electrode with Ni92% cathode (c) pristine electrode and (d) electrode with 2 wt% MWCNTs.
