# Supplementary material for: Improving Electrochemical Performance of Ultrahigh-Loading Cathodes via the Addition of Multi-Walled Carbon Nanotubes
Source: Nanomaterials (Basel). 2025 Jan 21;15(3):156. doi: 10.3390/nano15030156 (PMC11820175; doi:10.3390/nano15030156)
Supplement: Supplementary file 1 [file nanomaterials-15-00156-s001.zip › nanomaterials-3385083-supplementary.pdf]

Supplementary Materials

Improving Electrochemical Performance of Ultrahigh-Loading Cathodes via the Addition of Multi-Walled Carbon Nanotubes

Chan Ju Choi[a], Tae Heon Kim[a], Hyun Woo Kim[b], Do Man Jeon[c,*] and Jinhyup Han[a,*]

a. Department of Chemical Engineering, Keimyung University, Daegu 42601, Republic of Korea

b. Research Center for Materials Analysis, Korea Basic Science Institute (KBSI), Daejeon 34133, Republic of Korea

c. Gumi Electronics & Information Technology Research Institute (GERI), Gumi 39171, Republic of Korea

*Corresponding authors: jhan@kmu.ac.kr (J. Han) & jdm1213@geri.re.kr (D.M. Jeon)

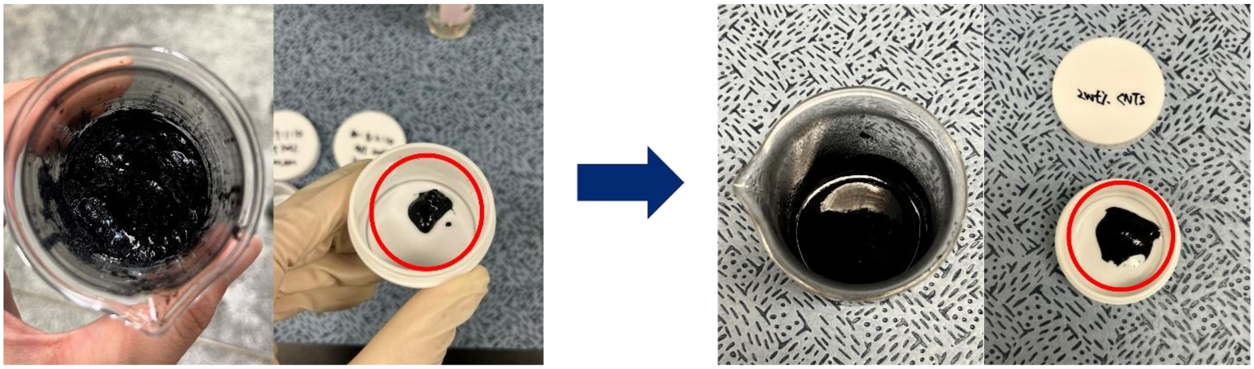

< Normal sonicator > **< Ultrasonication >**

Figure S1. Comparison of MWCNTs dispersion using normal sonication and ultrasonication. MWCNTs are homogenously distributed through ultrasonication.

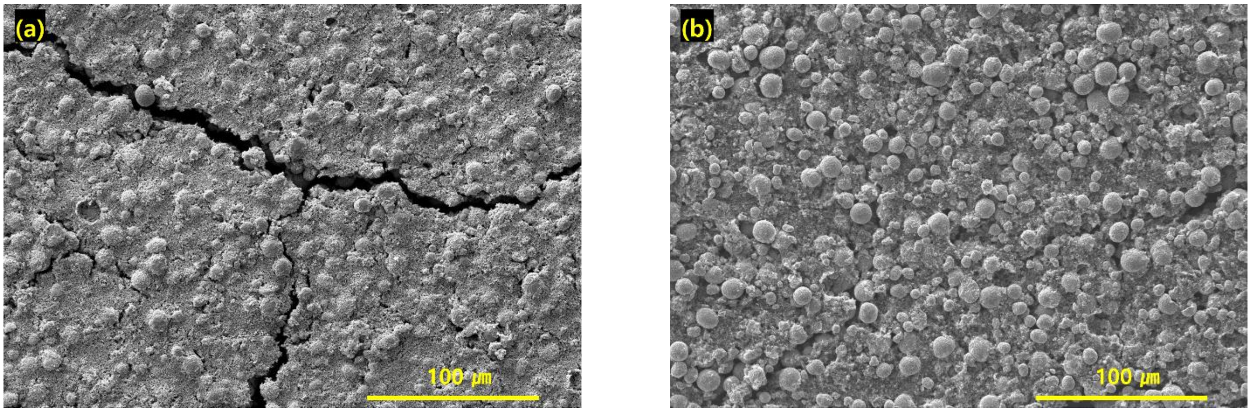

Figure S2. Surface images of electrodes after conducting C-rate capability test, performing recovery from high current rate of 1 C to lower rate of 0.1 C for (a) pristine cathode and (b) MWCNT-incorporated cathode.

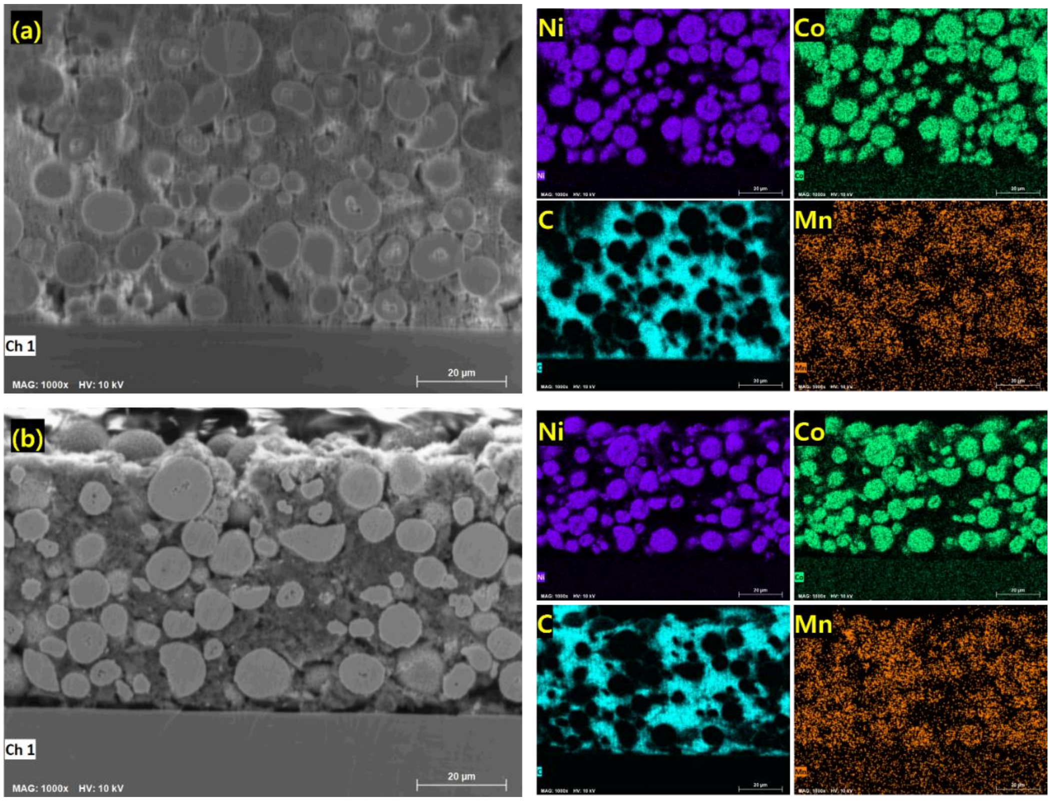

Figure S3. Energy dispersive spectroscopy mapping images of NCM811 cathode metals in the internal structure of the (a) electrode containing only carbon black and (b) electrode incorporating 2 wt% MWCNTs.

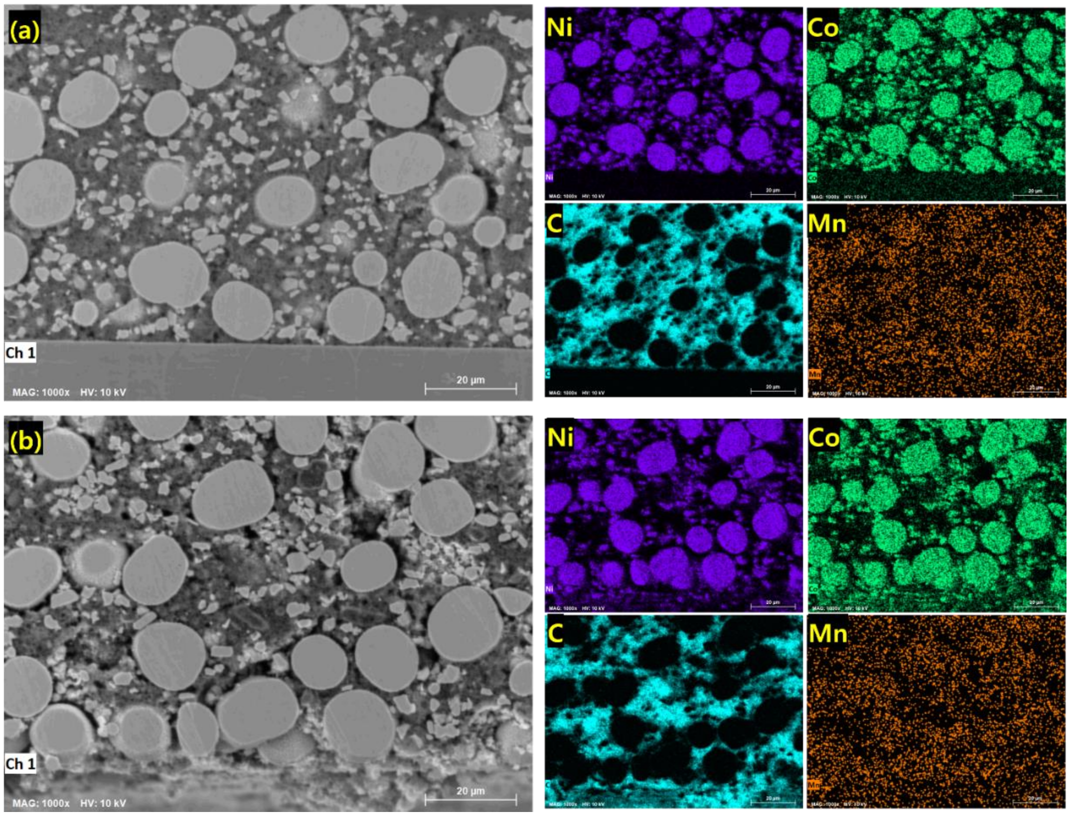

Figure S4. Energy dispersive spectroscopy mapping images of Ni92% cathode metals in the internal structure of the (a) electrode containing only carbon black and (b) electrode incorporating 2 wt% MWCNTs.

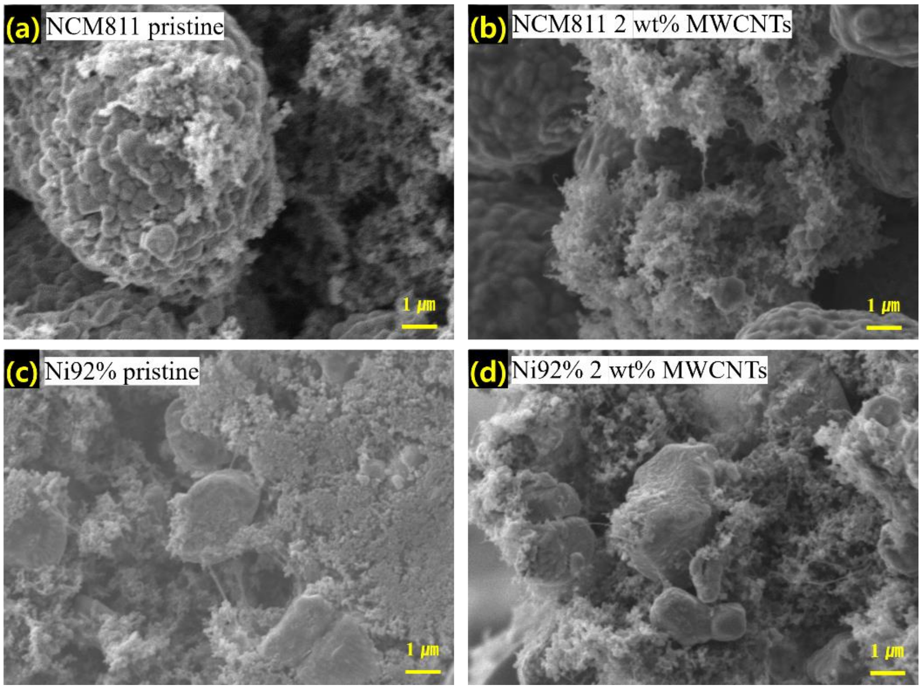

Figure S5. Images of inside the electrode with NCM811 (a) pristine electrode and (b) electrode with 2 wt% MWCNTs. Images of inside the electrode with Ni92% cathode (c) pristine electrode and (d) electrode with 2 wt% MWCNTs.
